# Cost Analysis of Recurrent Emergency Department Visits Among Patients Aged 65 and Older: A Retrospective Cross-Sectional Study

**DOI:** 10.7759/cureus.82966

**Published:** 2025-04-25

**Authors:** Teoman Ersen, Özhan Özcan, Kıvanç Öncü, Servan Gokhan

**Affiliations:** 1 Department of Emergency, Sinop Atatürk State Hospital, Sinop, TUR; 2 Department of Critical Care Medicine, Ege University Faculty of Medicine, Izmir, TUR; 3 Department of General Surgery, Istanbul Education Research Hospital, Istanbul, TUR; 4 Department of Anaesthesia and Reanimation, Sinop Atatürk State Hospital, Sinop, TUR; 5 Department of Emergency, Ankara Yildirim Beyazit University, Ankara, TUR

**Keywords:** cost analysis, emergency care, frail elderly, healthcare costs, hospital readmission

## Abstract

Introduction

This study aimed to analyze the costs of emergency department (ED) visits among patients aged 65 years and older, with a particular focus on the financial burden of recurrent admissions within a one-year period.

Methods

A retrospective cross-sectional study was conducted on 143,909 ED visits recorded between January 1, 2014, and December 31, 2014, at the Emergency Department of Ankara Atatürk Training and Research Hospital, Ankara, Turkey. Data for patients aged 65 and older were extracted from the Hospital Information Management System. Cost data were based on the Social Security Institution billing system and converted into US dollars (USD) using the 2014 exchange rate. Patients were categorized by age, gender, diagnosis, and visit frequency. Nonparametric statistical tests were used due to the non-normal distribution of cost variables. A p-value < 0.05 was considered statistically significant.

Results

A total of 21,458 (15.0%) ED visits were made by patients aged 65 and older. The median cost per visit in this group was $58.16. Costs increased with age: $42.90 for patients aged 65-74, $76.67 for those aged 75-84, and $96.42 for those aged ≥85 (Kruskal-Wallis H = 1,125.3, df = 2, p < 0.001). Among the 19,159 elderly patients who visited the ED, 1,951 (10.2%) had recurrent visits. Within this subgroup, internal medicine diagnoses were most common (1,345 visits, or 68.9%), followed by pulmonary (320, or 16.4%), cardiovascular (211, or 10.8%), and non-specific complaints (231, or 11.8%) as the leading causes. This diagnostic distribution differed significantly from that of single-visit patients (χ² = 42.7, df = 3, p < 0.001). Recurrence rates varied significantly by diagnostic category (χ² = 89.4, df = 5, p < 0.001): the highest recurrence was observed in patients with hematologic conditions (56/355, or 15.8%; adjusted OR = 1.72, 95% CI: 1.28-2.31), followed by psychiatric (8/57, or 14.0%; adjusted OR = 1.45, 95% CI: 0.99-2.12) and pulmonary diagnoses (320/2,358, or 13.6%; adjusted OR = 1.38, 95% CI: 1.22-1.57), all above the overall recurrence rate of 10.2%. The median cost of the first ED visit was significantly higher in the recurrent group ($72.13) compared to the non-recurrent group ($59.76) (Mann-Whitney U = 14.2 × 10⁶, p < 0.001, r = 0.14). Among recurrent cases, the mean cost of the first visit ($101.84) exceeded the average cost of subsequent visits ($93.98) (Wilcoxon T = 2.4 × 10⁵, p < 0.001, r = 0.09).

Conclusion

Older patients generate disproportionately higher ED costs in Turkey, particularly those with recurrent visits and chronic conditions. These findings support the implementation of geriatric-focused emergency care models and preventive strategies to optimize resource utilization in aging populations.

## Introduction

The global elderly population is growing at an unprecedented rate, and Turkey is no exception to this demographic shift. Aging is a natural phase of life, and the number of elderly individuals worldwide is projected to rise from 580 million in 1998 to 1.97 billion by 2050 [1]. This increase in life expectancy has led to a growing elderly population, necessitating greater attention to the challenges faced by older adults, particularly in healthcare. Identifying and understanding the health challenges associated with aging is essential for designing effective and sustainable healthcare services [2-6].

Aging is a multifactorial biological process characterized by cellular senescence, genomic instability, and mitochondrial dysfunction, leading to a progressive decline in physical and mental capacities, diminished homeostasis, and overall deterioration in health. Elderly individuals exhibit increased susceptibility to stressors and environmental changes, resulting in a higher prevalence of chronic diseases and multimorbidity. Consequently, they frequently utilize healthcare services, leading to elevated healthcare costs. The physiological changes associated with aging - such as atypical disease presentations, altered pharmacodynamics, reduced functional reserves, and social challenges - complicate the clinical evaluation and management of elderly patients in the emergency department (ED). These complexities often necessitate extended assessment times and increased resource utilization. Additionally, communication barriers between healthcare providers and elderly patients or their caregivers further hinder effective care delivery [7-10].

Elderly patients are more likely to visit the ED due to the higher prevalence of chronic diseases, polypharmacy, and atypical disease presentations, which complicate their clinical management. The number of ED visits by individuals aged 65 and older continues to rise annually, with variations influenced by factors such as country, city, location of the ED, and the demographic characteristics of the population [11-15]. According to the U.S. National Center for Health Statistics (NCHS) 2011 report, 36.9% of elderly ED visits were by patients aged 65-74, while 58.2% were by those aged 75 and older [16].

Elderly patients visit the ED and healthcare facilities for a variety of reasons, leading to increased demand for healthcare services. This, in turn, draws attention to healthcare costs. In many developed countries, cost-containment models are being developed to control healthcare expenditures. However, standardizing institutional healthcare services is a complex and lengthy process [11,12,14,17].

Cost studies are essential for delivering healthcare services more efficiently and effectively to the population. Studies that assess the economic burden of diseases on society are referred to as "cost of illness" studies. Additionally, there are various cost analyses that compare different drugs and treatment approaches, such as cost-benefit, cost-utility, and cost-effectiveness analyses [18,19].

In Turkey, cost analysis studies remain limited due to the lack of standardized data collection methods and the complexity of integrating healthcare expenditure data from multiple sources [20-22]. Disease-related costs are typically divided into direct healthcare expenditures - covering diagnosis, treatment, medication, diagnostic tests, follow-up, outpatient and emergency care, and hospitalizations - and indirect healthcare expenditures, which arise from productivity losses due to illness, disability, or premature death. Among direct costs, hospitalization represents the largest expenditure, while indirect costs primarily stem from reduced productivity and activity limitations [18,19,23,24].

Given the significant economic burden of aging-related healthcare utilization, this study aims to analyze the costs and frequency of ED visits among elderly patients in Turkey, with a focus on healthcare expenditures.

## Materials and methods

This study was designed as a retrospective cross-sectional analysis. Data were retrospectively collected from the Hospital Information Management System (HIMS) at Ankara Atatürk Training and Research Hospital, Ankara, Turkey, encompassing a total of 143,909 ED visits between January 1, 2014, and December 31, 2014. The study specifically focused on ED visits by patients aged 65 years and older, including both single and recurrent admissions. Patients with incomplete or missing records, those presenting solely for routine check-ups, and cases without cost data were excluded from the analysis. All patient data were anonymized, and confidentiality was strictly maintained in compliance with institutional and ethical guidelines.

Patient costs were calculated using the direct cost system, which included expenses billed to the Social Security Institution. These costs encompassed consultation fees, laboratory tests, radiological imaging, medication costs, and hospitalization expenses. Indirect costs, such as loss of workforce, disability-related expenses, and hospital staff salaries, were excluded from the analysis. To standardize the cost data, all expenses were converted into US dollars (USD) using the exchange rate of 2.19 TRY/USD, as published by the Central Bank of the Republic of Turkey on January 1, 2014.

Statistical analyses were performed using IBM SPSS Statistics for Windows, Version 22 (Released 2013; IBM Corp., Armonk, NY, USA). Categorical variables were summarized using frequency distributions, while continuous variables were presented as descriptive statistics (mean, median, standard deviation, etc.). The Kolmogorov-Smirnov test was applied to assess the normality of continuous variables. Since the normality assumption was not met (p < 0.05), nonparametric tests were used for comparisons. The Mann-Whitney U test was employed for comparing independent groups, while the Chi-square test was used to analyze independent dichotomous variables. For paired sample comparisons, the Wilcoxon test was applied, and the Kruskal-Wallis test was used for multiple group comparisons, followed by Bonferroni correction when necessary. A p-value < 0.05 was considered statistically significant in all analyses.

## Results

**Figure 1:**
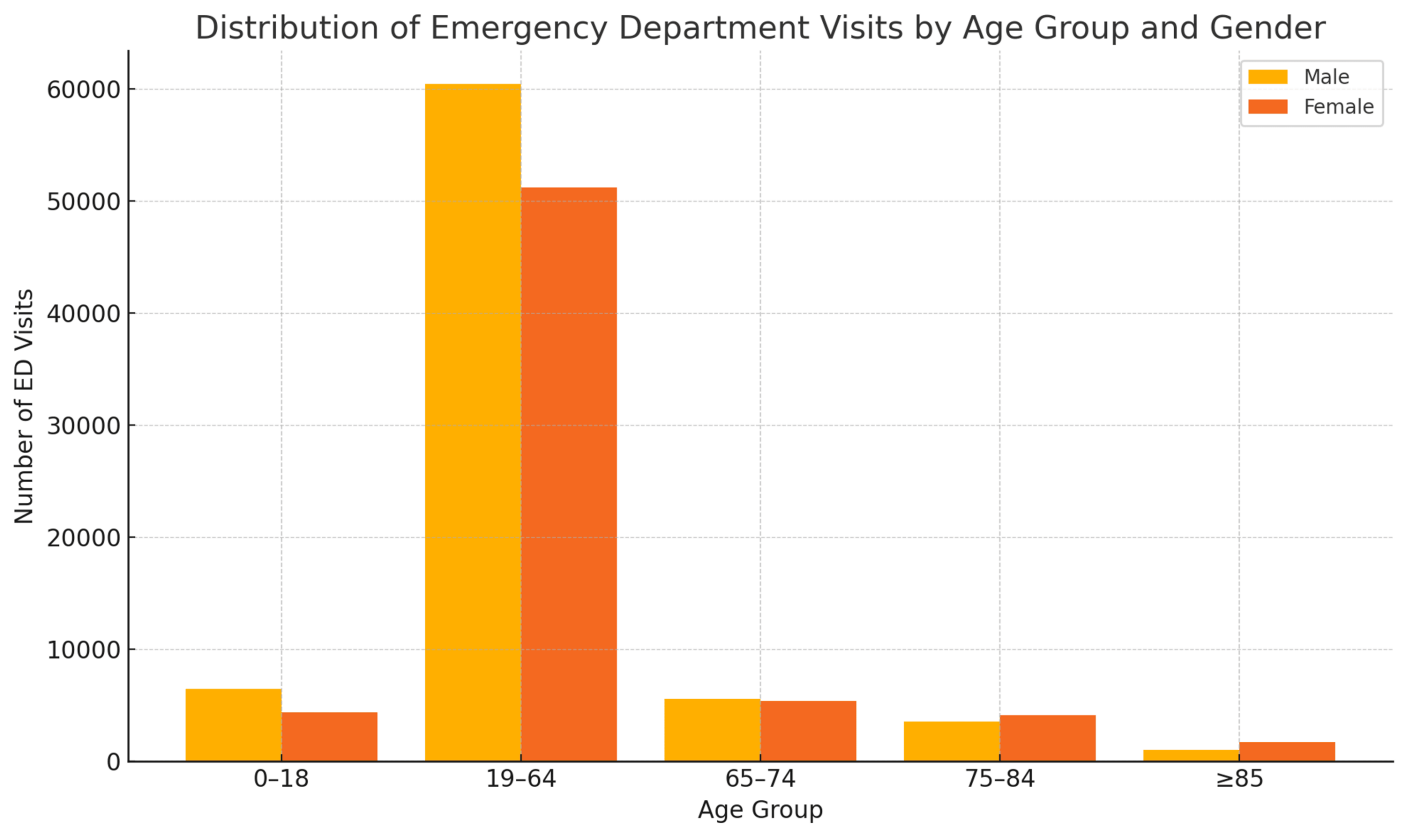
Distribution of Emergency Department (ED) Visits by Age Group and Gender (N = 143,909)

During the one-year study period, a total of 143,909 ED visits were recorded (Table 1 and Figure 1). Among these, 21,458 (15%) were made by individuals aged 65 years and older. A gender-based breakdown of these elderly ED visits revealed that 10,216 (47.6%) were by males and 11,242 (52.4%) by females. Age group distribution within this cohort indicated that 51% (10,951) of visits were made by patients aged 65-74 years, 36% (7,710) by those aged 75-84 years, and 13% (2,797) by those aged ≥85 years. Notably, in the ≥85 age group, 64.2% (n = 1,745, χ²(1) = 18.3, p < 0.001, φ = 0.09) of visits were made by female individuals, underscoring a gender-related shift in healthcare utilization in the oldest age bracket. Additionally, the median age associated with ED visits was significantly higher in females compared to males (40 vs. 37 years, χ²(1) = 42.7, p < 0.001, Cramer's V = 0.15, p < 0.001).

**Table 1:** Demographic Distribution of All ED Visits by Age Group and Gender *Gender difference in median age: p < 0.001 ED, emergency department

Age groups, n (%)	n		% in age group		% in gender group	n		% in age group		% in gender group
Total (n = 143,909)	Male: n = 77,100 (53.6%), Median age: 37*					Female: n = 66,809 (46.4%), Median age: 40*				
0-18 (10,855, 7.5%)	6,471	59.6		8.4		4,384	40.4		6.6	
19-64 (n = 111,569, 77.5%)	60,413	54.1		78.4		51,183	45.9		76.6	
≥65 (total = 21,458, 15.0%)	10,216	47.6		13.2		11,242	52.4		16.8	
65-74 (n = 10,951, 51%)	5,581	51		54.6		5,370	49		47.8	
75-84 (n = 7,710, 36%)	3,583	46.5		35.1		4,127	53.5		36.7	
≥85 (n = 2,797, 13%)	1,052	37.6		10.3		1,745	64.2		15.5	

A total of 19,159 (89.8%) individual patients aged 65 and over were identified among the elderly visit cohort (Table 2). Of these, 1,951 patients (10.2%) presented to the ED more than once during the study period, while 17,208 (89.8%) had a single visit. Recurrent ED visits were more frequent among older subgroups: 9.6% (941/9,789) in the 65-74 age group, 10.9% (746/6,862) in the 75-84 age group, and 10.5% (264/2,508) in those aged ≥85 years (p = 0.012). Gender distribution within this subgroup showed similar recurrence rates for males (9.6%) and females (9.9%) (χ²(1) = 0.8, p = 0.36, φ = 0.02).

**Table 2:** Distribution of Repeat ED Visits Among Elderly (≥65 Years) Patients by Age and Gender ED, emergency department

Total (n = 191,59)	No repeat visit (n = 17,208, 89.8%)			Repeat visit (n = 1,951, 10.2%)		
	n (%)	% in group	% of total	n (%)	% in group	% of total
Gender						
Male (n = 9,051, 47.2%)	8,104 (47.1%)	90.4%	42.3%	947 (48.5%)	9.6%	4.9%
Female (n = 10,108, 52.8%)	9,104 (52.9%)	90.5%	47.5%	1,004 (51.5%)	9.9%	5.2%
Age groups						
65-74 (n = 9,789, 51.1%)	8,848 (51.4%)	90.4%	46.2%	941 (48.2%)	9.6%	4.9%
75-84 (n = 6,862, 35.8%)	6,116 (35.5%)	89.1%	31.9%	746 (38.2%)	10.9%	3.9%
≥85 (n = 2,508, 13.1%)	2,244 (13.1)	89.5%	11.7%	264 (13.5%)	10.5%	1.4%

Diagnostic patterns differed significantly between single and repeat visitors (p < 0.001 for all comparisons) (Table 3). Non-recurrent visits were predominantly for internal medicine conditions (11,446 visits, or 66.5%), non-traumatic surgical diagnoses (3,829, or 22.3%), and trauma-related presentations (1,888, or 11.0%). Among repeat visitors, internal medicine diagnoses accounted for 1,345 visits (68.9%), with higher proportions of pulmonary (320, or 16.4%), cardiovascular (211, or 10.8%), and non-specific complaints (231, or 11.8%). Hematologic (15.8%, or 56/355 visits) and psychiatric (14%, or 8/57 visits) diagnoses were overrepresented in the repeat visit group, whereas trauma-related visits were less frequent (6.3%, or 127/2,015 visits).

**Table 3:** Distribution of Initial Emergency Diagnoses in Elderly (≥65 Years) Patients, With and Without Repeat Visits

All initial visits (n = 19,159)	No repeat visit (n = 17,208)			Repeat visit (n = 1,951)		
	n (%)	% within diagnosis group	% of all patients	n (%)	% within diagnosis group	% of all patients
Non-traumatic surgical diagnoses (n = 4,308, 22.5%)	3,829 (22.3)	88.9	19.99	479 (24.6)	11.1	2.50
Abdominal surgical (n = 1,969, 10.3%)	1,746 (10.1)	88.7	9.11	223 (11.4)	11.3	1.16
Non-traumatic orthopedic (n = 1,750, 9.1%)	1,559 (9)	89.1	8.14	191 (9.8)	10.9	1.00
Urological (n = 584, 3.0%)	520 (3)	89	2.71	64 (3.3)	11	0.33
Obstetrics and gynecology (n = 5, 0.02%)	4 (0.02)	80	0.02	1 (0.01)	20	0.01
Internal medicine diagnoses (n = 12,791, 66.7%)	11,446 (66.5)	89.5	59.74	1,345 (68.9)	10.5	7.02
Pulmonary (n = 2,358, 12.3%)	2,038 (11.8)	86.4	10.64	320 (16.4)	13.6	1.67
Non-specific complaints (n = 2,137, 11.2%)	1,906 (11.1)	89.2	9.95	231 (11.8)	10.8	1.21
Cardiac (n = 2,024, 10.6%)	1,813 (10.5)	89.6	9.46	211 (10.8)	10.4	1.10
Gastrointestinal (n = 1,371, 7.2%)	1,223 (7.1)	89.2	6.38	148 (7.6)	10.8	0.77
Neurological/Non-Surgical Neurosurgical (n = 1,909, 10.0%)	1,763 (10.2)	92.4	9.20	146 (7.5)	7.6	0.76
Infectious diseases (n = 1,022, 5.3%)	919 (5.3)	89.9	4.80	103 (5.3)	10.1	0.54
ENT (Ear-Nose-Throat) (n = 916, 4.8%)	850 (4.9)	92.8	4.44	66 (3.4)	7.2	0.34
Hematologic (n = 355, 1.9%)	299 (1.7)	84.2	1.56	56 (2.9)	15.8	0.29
Nephrological (n = 203, 1.1%)	180 (1)	88.7	0.94	23 (1.2)	11.3	0.12
Dermatologic (n = 231, 1.2%)	213 (1.2)	92.2	1.11	18 (0.9)	7.8	0.09
Peripheral vascular (n = 79, 0.4%)	70 (0.4)	88.6	0.37	9 (0.5)	11.4	0.05
Psychiatric (n = 57, 0.3%)	49 (0.3)	86	0.26	8 (0.4)	14.0	0.04
Endocrinological (n = 116, 0.6%)	110 (0.6)	94.8	0.57	6 (0.3)	5.2	0.03
Toxicological (n = 13, 0.1%)	13 (0.08)	100	0.07	0 (0)	0	0.00
Trauma related diagnoses (n = 2,015, 10.5%)	1,888 (11.0)	93.7	9.85	127 (6.5)	6.3	0.66
Burns (n = 15, 0.1%)	15 (0.1)	100	0.08	0 (0)	0	0.00
Trauma (n = 2,000, 10.4%)	1,873 (10.9)	93.6	9.78	127 (6.5)	6.4	0.66
Cardiac arrest (n = 45, 0.2%)	45 (0.3)	100	0.23	0 (0)	0	0.00

Cost analysis demonstrated a clear association between increasing age and rising ED expenditure (Table 4). The median cost per visit was $14.46 for individuals aged 0-18 years, $15.12 for those aged 19-64 years, and markedly higher at $58.16 for the ≥65 age group (Kruskal-Wallis H = 9,842, p < 0.001, η² = 0.28). Within the elderly population, the median cost increased progressively with age: $42.90 for the 65-74 age group, $76.67 for the 75-84 group, and $96.42 for those aged ≥85 (Kruskal-Wallis H = 1,125.3, df = 2, p < 0.001).

**Table 4:** Cost Analysis (USD) of Emergency Department Admissions by Age and Diagnosis Groups SD, standard deviation

Age Groups	Mean ($)	Median ($)	SD	Min ($)	Max($)	p
0-18 (n = 10,855, 7.5%)	27.94	14.46	34.48	7.07	642.82	0.001
19-64 (n = 111,569, 77.5%)	35.50	15.12	83.48	7.07	9,738.92	<0.001
≥65 total (n = 21,458, 15.0%)	90.13	58.16	269.08	7.07	26,048.13	<0.001
≥65 first visits (n = 19,159)	-	61.43	-	7.07	6,497.3	-
65-74 (n = 9,789)	-	42.90	-	7.07	3,380.64	<0.001
75-84 (n = 6,862)	-	76.67	-	7.07	6497.03	<0.001
≥85 (n = 2,508)	-	96.42	-	7.07	1674.26	<0.001
Diagnosis-based groups (n = 19,114)	-	61.15	-	7.07	6497.03	-
Non-traumatic surgical diagnoses (n = 4,308)	-	42.72	-	7.07	1,414.12	<0.001
Internal medicine diagnoses (n = 12,791)	-	69.10	-	7.07	6,497.03	<0.001
Trauma diagnoses (n = 2,015)	-	62.52	-	7.07	2,836.07	0.775

Among diagnostic categories, internal medical conditions incurred the highest median costs ($69.10), followed by trauma-related conditions ($62.52) and non-traumatic surgical diagnoses ($42.72) (χ²(2) = 893, p < 0.001, η² = 0.18) (Table 4). When comparing overall patients with and without recurrent visits, the median cost of the first ED presentation was significantly higher in the recurrent group ($72.13 vs. $59.76; Mann-Whitney U = 14.2 × 10⁶, p < 0.001, r = 0.14) (Table 5). This pattern was consistent across gender and age strata. For instance, among female patients, the median cost of the initial visit in the recurrent group was $75.70, compared to $61.13 in the non-recurrent group (U = 7.1 × 10⁶, p < 0.001, r = 0.16). In the 65-74 age group, corresponding figures were $57.48 and $41.51, respectively (U = 3.8 × 10⁶, p < 0.001, r = 0.18).

**Table 5:** Cost Distribution (USD) of First ED Admissions in Elderly Patients by Revisit Status, Gender, Age, and Diagnosis ED, emergency department

All patients (n = 19,159)	No revisit (n = 17,208; 89.8%): $59.76 (min-max: $7.07-$6,494.04)		Revisit (n = 1,951; 10.2%): $72.13 (min-max: $7.07-$3,380.64)		p < 0.001
	n	Median cost (min-max)	n	Median cost (min-max)	p
Gender					
Male (n = 9,051)	8,104	$58.49 (7.07-6,322.72)	947	$69.05 (7.07-3,380.64)	<0.001
Female (n = 10,108)	9,104	$61.13 (7.07-6,494.07)	1,004	$75.70 (7.07-2,330.98)	<0.001
Age groups					
65-74 (n = 9,789)	8,848	$41.51 (7.07-1,910.91)	941	$57.48 (7.07-3,380.64)	<0.001
75-84 (n = 6,862)	6,116	$75.75 (7.07-6,494.07)	746	$82.46 (7.07-1,581.66)	0.007
≥85 (n = 2,508)	2,244	$95.80 (7.07-1,673.49)	264	$102.29 (7.07-1,028.28)	0.073
Diagnoses					
Non-traumatic surgical (n = 4,308)	3,829	$42.28 (7.07-1,413.48)	479	$47.45 (7.07-1,186.23)	0.025
Internal medicine (n = 12,791)	11,446	$67.52 (7.07-6,494.07)	1,345	$80.91 (7.07-3,380.64)	<0.001
Trauma (n = 2,015)	1,888	$61.58 (7.07-2,834.79)	127	$76.18 (7.07-658.22)	0.091

Among elderly patients with recurrent ED admissions (n = 1,951), a comparison was made between the cost of their first visit and the average cost of all subsequent visits (Table 6). The mean cost of the initial ED visit was calculated as $101.84, while the mean cost averaged across all ED visits in this group was $93.98 (Wilcoxon T = 2.4 × 10⁵, p < 0.001, r = 0.09). Although both figures indicate substantial healthcare expenditures, the initial visit incurred significantly higher costs than the subsequent ones.

**Table 6:** Comparison of Initial and Average Costs (USD) of Repeat ED Visits in Elderly Patients SD, standard deviation; ED, emergency department

	n	Mean ($)	Median ($)	SD	Minimum ($)	Maximum ($)	p
Initial visit cost of repeat visitors	1,951	101.8373	72.1324	143.5394	7.07	3,380.64	<0.001
Average cost of repeat visits per patient	1,951 (2,299)	93.9827	71.8849	106.9146	7.07	2,419.8995	<0.001

## Discussion

This study provides comprehensive insight into ED utilization patterns and cost distribution among individuals aged 65 years and older. The findings confirm that elderly patients account for a considerable share of ED visits and incur significantly higher healthcare costs, particularly in cases of recurrent admissions. These outcomes reflect the broader demographic trend of population aging and the growing burden it places on emergency services worldwide [1,2].

The predominance of elderly female patients, especially in the ≥85 age group, aligns with global data demonstrating longer life expectancy and higher healthcare utilization among older women [2,3]. This may be attributed to the increased prevalence of frailty, functional decline, and social vulnerability in older females [4,25].

Approximately 10.2% of elderly patients in our cohort had recurrent ED visits within a single year. These repeat admissions were more frequently associated with internal medical conditions, such as pulmonary and cardiologic diagnoses, as well as nonspecific complaints. These findings are consistent with previous studies showing that multimorbidity and geriatric syndromes - including cognitive impairment, falls, and functional limitations - are strong predictors of ED readmission and poor postdischarge outcomes [5,6,25]. In particular, frailty has been identified as a key factor associated with increased ED visits and hospital revisits among older adults [26,27].

Moreover, recurrent admissions were also more common in patients with psychiatric and hematologic diagnoses, suggesting that fragmented care and limited outpatient support for complex conditions contribute to repeat ED use. These observations reinforce the need for more integrated geriatric care across healthcare settings [13,14].

In terms of cost, our analysis showed a significant increase in ED visit expenditures with advancing age, consistent with prior literature highlighting the resource-intensive nature of care for older patients [7,11]. Internal medical diagnoses were associated with the highest median costs, likely reflecting the need for comprehensive diagnostic workups and prolonged clinical evaluation. Notably, among patients with recurrent ED visits, initial presentations incurred higher average costs than subsequent visits - possibly due to more severe symptomatology or the need for stabilization.

To address the clinical and economic challenges posed by this population, evidence supports the implementation of structured geriatric interventions. Comprehensive geriatric assessments, transitional care planning, and case management have been shown to reduce ED revisits and improve outcomes for vulnerable elderly patients [4,28]. Successful models, such as geriatric emergency networks, also demonstrate the effectiveness of interdisciplinary approaches in reducing unplanned readmissions and enhancing care continuity [28].

In addition, a redefinition of the ED’s role in the healthcare system has been proposed, advocating for EDs to serve not only as acute care providers, but also as strategic access points for identifying at-risk older adults and initiating multidisciplinary care pathways [29]. Incorporating frailty screening and discharge planning into ED protocols could help reduce future admissions and enhance cost-efficiency [26,27].

From a health policy perspective, the findings of this study underscore the value of cost-of-illness analyses in aging populations. Accurate identification of high-cost, high-risk subgroups - such as elderly patients with recurrent admissions - can inform the design of targeted, resource-efficient interventions [18,19].

Limitations

This study has several limitations. First, its retrospective, single-center design may limit generalizability to other institutions or health systems [13]. Second, only direct medical costs were analyzed; indirect costs, such as caregiver burden, productivity loss, or long-term disability, were not included, which may underestimate the full economic impact [19]. Third, clinical variables such as comorbidities, frailty, or functional capacity were unavailable, despite their known influence on emergency care outcomes in older adults [25,27]. Lastly, healthcare delivery models, insurance structures, and reimbursement systems vary considerably across countries, which may influence both patterns of ED utilization and associated costs. Therefore, these findings may not be directly generalizable to other international settings [18].

## Conclusions

Elderly individuals represent a growing and high-risk population in EDs, contributing disproportionately to healthcare utilization and costs. This study highlights the significant association between advanced age, recurrent ED admissions, and elevated medical expenditures - particularly among patients with internal medical diagnoses and geriatric syndromes. The findings support the need for integrated, geriatric-focused interventions, such as comprehensive assessments, transitional care models, and frailty screening, to reduce avoidable ED visits and improve healthcare efficiency in aging populations. These strategies are essential not only to optimize clinical outcomes but also to ensure the sustainable use of emergency care resources in the face of global population aging.
